# Water Matters More: Unequal Effects of Water and Sanitation on Child Growth in Mozambique

**DOI:** 10.3390/children12101414

**Published:** 2025-10-20

**Authors:** Jailene P. Castillo, Christina A. Molinaro, William E. Pater, Santosh K. Gautam

**Affiliations:** 1 Keough School of Global Affairs, University of Notre Dame, Notre Dame, IN 46556, USA; jpcas021@gmail.com; 2 Eck Institute for Global Health, University of Notre Dame, Notre Dame, IN 46556, USA; 3 Department of Biological Sciences, University of Notre Dame, Notre Dame, IN 46556, USA

**Keywords:** child, stunting, wasting, water, sanitation, Mozambique

## Abstract

**Highlights:**

**What are the main findings?**

• Despite WASH interventions, 33.1% and 46.5% of children under five were stunted and wasted in Mozambique, respectively.
• Improved water sources are more protective against stunting than wasting.

**What is the implication of the main finding?**

• Improved sanitation is moderately effective against wasting compared to stunting.
• WASH interventions cannot be applied uniformly to address both stunting and wasting.

**Abstract:**

**Background**: Child stunting and wasting persist at alarmingly high rates in Mozambique, yet little is known about whether the improved sources of water and sanitation affect these outcomes differently. This study aims to disentangle the distinct contributions of improved water sources and sanitation facilities to child stunting and wasting at the national level, addressing a critical evidence gap in the WASH–nutrition literature in Mozambique. **Methods**: Using data from 3690 children under five in the 2022–2023 Mozambique Demographic and Health Survey, we applied stepwise logistic regression models to estimate the independent and combined associations of improved drinking water and sanitation facilities with child stunting and wasting, adjusting for child-, household-, and region-level factors. **Results**: Improved water access was significantly associated with a lower risk of stunting (odds ratio = 0.80, 95% CI: 0.67–0.94, *p* < 0.01), while sanitation showed only weak and inconsistent associations with stunting. In the fully adjusted model, neither improved water nor sanitation was associated with wasting. Wealth, gender, religion, and region were also significant predictors of stunting as well as wasting. **Conclusions**: These findings indicate that WASH components protect against child malnutrition through different pathways, with water being more protective against chronic undernutrition and sanitation less clearly linked to acute malnutrition. Broader socioeconomic and cultural factors—such as wealth, religion, and geography—play critical roles, highlighting the need for integrated, context-specific interventions.

## 1. Introduction

Malnutrition among children continues to persist as a major global health challenge, particularly in low- and middle-income countries. Among children, the two primary manifestations of malnutrition are stunting and wasting. Stunting (low height-for-age, >2 SD below the WHO Growth Standards median) reflects chronic undernutrition and repeated infection, while wasting (low weight-for-height) signals acute undernutrition. In 2024, 150.2 million (23.2%) under-five children were stunted, and 42.8 million (6.5%) were wasted, reflecting chronic growth faltering and acute undernutrition [1]. Stunting and wasting prevalence vary significantly by region, with South Asia and Sub-Saharan Africa consistently reporting the highest rates of stunting. In Sub-Saharan Africa, the burden is higher: roughly one in three children is stunted (35%), and about 6% are wasted. The major risk factors for child malnutrition include poor diets and food insecurity; inadequate maternal nutrition and health; infectious disease burdens; unsafe water, sanitation, and hygiene (WASH); poverty and suboptimal childcare; and limited access to quality health services. These determinants can interact and reinforce one another: acute wasting episodes can slow linear growth, while chronically stunted children are more vulnerable to wasting during shocks. Among these factors, the WHO highlights WASH as essential to preventing malnutrition. WASH influences nutrition through the prevention of diarrheal disease, environmental enteric dysfunction, and impaired nutrient absorption [2,3]. Yet inadequate WASH remains widespread: nearly two billion people lack access to safe drinking water, and 3.6 billion lack access to safe sanitation services worldwide [4].

Higher prevalence of malnutrition among children has considerable economic, health, and human costs. Biologically, undernutrition weakens immunity and raises the risk and severity of infections, contributes to maternal and neonatal complications, and increases child mortality; early-life deficits also impair brain development, with largely irreversible effects on cognition. These health shocks translate into fewer years of schooling, lower test scores, and diminished skills, which depress adult productivity and wages. Globally, the annual economic cost of the current level of stunting is $548 billion, equivalent to 0.7% of the global income [5]. The annual human capital costs include 1.3 million deaths, 304 million IQ points lost, and 49 million school years lost globally [5]. Undernutrition contributes to up to 45% of preventable under-five deaths; severely undernourished children face up to a nine-fold higher risk of death than well-nourished peers [6].

A growing body of research highlights the role of WASH in shaping child nutrition outcomes, yet the evidence is mixed. Evidence from Indonesia and multi-country cohort studies from the Young Lives survey suggests that improved water and sanitation can reduce stunting, especially when combined interventions are adopted [7,8]. However, large-scale randomized trials in Zimbabwe, Kenya, and Bangladesh have shown limited effects of household-level WASH programs on child growth, indicating that stunting may reflect more chronic exposures and structural factors [3,9,10]. For wasting, the evidence is weaker; most studies show little consistent association between WASH and acute malnutrition, though sanitation improvements have occasionally been linked to lower odds of wasting [11,12]. Taken together, these studies suggest that while WASH can be protective, its effects differ for stunting versus wasting and depend heavily on context and complementary interventions. Against the mixed evidence and persistent regional burdens, we turn to Mozambique, where large WASH gaps coincide with high stunting and persistent wasting; this setting allows us to test whether water and sanitation are differentially associated with these outcomes in a nationally representative sample.

**Figure 1:**
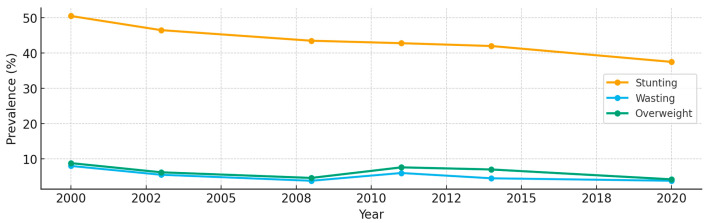
Child wasting and stunting in Mozambique from 2002 to 2020 [1].

WASH shortfalls and high child undernutrition constitute a dual crisis in Mozambique: only 56% of the population has basic drinking water, and 31% have basic sanitation [13]. In 2022, 37% of under-fives were stunted and 4% were wasted [14]; after declines from 2000 to 2020, progress has since plateaued (Figure 1). Stunting prevention requires sustained, multisectoral action, whereas wasting management targets acute episodes [15]. The economic toll of malnutrition in Mozambique is substantial—nearly 10% of GDP (≈US$124 million annually) in lost productivity and higher health spending [16,17]. Existing evidence is geographically narrow: in Maputo, shared sanitation is more strongly associated with stunting than with diarrhea [18]; in Beira, WASH access is highly uneven with limited analysis of downstream growth outcomes [19]; in southern Mozambique, undernutrition and diarrhea co-occur without a defined mechanism [20]; and sanitation upgrades in Maputo reduced enteric infection and diarrhea but did not assess stunting or wasting [21].

Despite widespread recognition of WASH as a critical determinant of child growth, evidence on which components of WASH matter for stunting versus wasting remains limited and context-dependent. Prior studies suggest water quality potentially being more relevant for acute conditions (diarrhea/enteric infection) and sanitation as more closely tied to chronic growth faltering, yet Mozambique lacks comprehensive analysis of nationally representative data, with the few existing studies exhibiting limitations in terms of scope, sample size, or variable definitions, limiting their generalizability.

The objective of this study is therefore to determine the associations between sources of drinking water and types of toilet facilities with child stunting and wasting in Mozambique. By analyzing these indicators both independently and jointly in a nationally representative dataset, we provide new evidence on the differentiated effects of water and sanitation. This distinction is scientifically significant because it helps clarify the specific pathways through which WASH conditions influence growth faltering, and clinically relevant because stunting and wasting carry distinct short- and long-term health consequences. By conducting a national analysis of differentiated WASH effects on distinct stunting and wasting outcomes, resources can be more effectively allocated to maximize potential positive impacts. From a policy perspective, by identifying whether water or sanitation has a stronger role in reducing stunting or wasting, resources can be allocated more efficiently and integrated into broader strategies to combat child malnutrition in Mozambique and similar low- and middle-income settings.

This study is the first to use nationally representative data from Mozambique to disentangle the distinct effects of water and sanitation on child stunting and wasting. By identifying these differential pathways, the study advances the WASH–nutrition literature and provides actionable evidence to guide more targeted and efficient policy interventions.

## 2. Materials and Methods

This is a quantitative, observational (cross-sectional) study using nationally representative survey data from Mozambique. We examine the association between access to improved drinking water and sanitation and children’s anthropometric outcomes (stunting and wasting).

### 2.1. Study Setting

Mozambique, located in southeastern Africa, covers approximately 801,590 square kilometers, making it the 35th largest country globally. As of 2025, the nation’s population is estimated at around 34 million, with a growth rate of 2.8% from the previous year [22]. The demographic is notably young, with over half of the population aged 19 and below. The life expectancy is approximately 57.1 years, and the under-five mortality rate improved to 66.21 per 1000 live births by 2023. As of 2022, the WHO estimated that just 63% of Mozambique’s population had access to basic water services, while only 38% had access to basic sanitation facilities. Additionally, around 29% of the population practiced open defecation nationwide.

### 2.2. Data Sources

The data used were obtained from the Demographic and Health Surveys (DHS) portal. It provides a comprehensive overview of Mozambique’s national health status in 2022–2023, covering a broad range of health topics like communicable and non-communicable diseases, nutrition, maternal and child health, and fertility and family planning, among others. This dataset used a two-stage stratified sampling design to ensure national and sub-national representativeness. Enumeration areas were selected using probability proportional to size, followed by systematic sampling of households within each cluster. Survey weights were applied to account for sampling design and non-response. A total of 14,250 households were sampled, of which the majority of respondents were females aged 15 to 49. From the total sample, we identified 26,207 children under the age of five. We excluded flagged cases, children with heights and weights out of plausible limits, and missing values. Our final analytic sample included 3690 children under the age of five. Disaggregated estimates were provided for ten regions: Niassa, Cabo Delgado, Nampula, Zambezia, Tete, Manica, Sofala, Inhambane, Gaza, and Maputo. The survey was implemented by Mozambique’s National Institute of Statistics in collaboration with the Ministry of Health and was technically supported by ICF International. Ethical approval was obtained by national authorities, and informed consent was secured from all participants.

### 2.3. Independent Variables

We use two main explanatory WASH indicators: the source of drinking water and the type of toilet facilities. These variables were recorded into binary outcomes showing improved = 1 and not improved = 0 WASH status according to the WHO’s classification.

### 2.4. Outcome Variables

Child undernutrition was assessed using two binary variables: stunting and wasting. Stunted children were coded as 1 and non-stunted as 0. The same applies to the wasting variable: wasted children were coded as 1, non-wasted as 0.

### 2.5. Control Variables

For each child under 5, we incrementally control variables that had previously been associated with child stunting and wasting globally and in Africa. First, we control for the child’s characteristics: gender (1 = female, 0 = male), age in months, and birth order. We proceed to include household socio-demographic factors like wealth index (1 being poorest and 5 being richest), religion, and household size. The Mozambique 2022–23 DHS constructs a household wealth index using principal components analysis applied to assets and living-standard indicators, then ranks households into five wealth quintiles (poorest to richest). Typical variables include in the wealth index are consumer durables (radio, television, refrigerator, mobile phone, computer, fan/air conditioner, washing machine); transport assets (bicycle, motorcycle/scooter, car/truck, plus country-specific items); housing quality (materials of floor, wall, and roof; crowding such as rooms per person); and utilities/services (electricity, main cooking fuel, source of drinking water, and type of toilet, including whether shared). We also factor in the regions and residence type, 1 being urban and 0 being rural. We include region fixed effects to account for the fixed characteristics of the regions that may affect the outcome variables. The regression model did not include the mother’s education due to the high frequency of missing values.

### 2.6. Statistical Analysis

We estimate sequentially adjusted logistic regression models to examine the association between access to improved sources of drinking water and sanitation and children’s anthropometric outcomes. The data was analyzed on the STATA/SE 18 software. We report odds ratios (OR) with 95% confidence intervals. Analyses were conducted in Stata/SE 18 (StataCorp, College Station, TX, USA). Stata was selected for its built-in support for complex survey analysis (svyset for weights, strata, and clusters), availability of logistic regression with robust standard errors, and postestimation tools (e.g., margins). Stata/SE also accommodates large, wide files typical of DHS data and facilitates a reproducible workflow using scripted do-files. The statistical estimators used in this study are standard and do not depend on the software platform. Model 1 assesses the correlation between the source of drinking water and child stunting alone. The same applies to model 2, except that the independent variable is the type of toilet facilities in the households. The third model considers the effect on child stunting by both the source of drinking water and the type of toilet facilities. Understanding that households may be confounded by other factors like socio-demographic and household characteristics, we measure the correlation between both WASH indicators and stunting in the presence of age, gender, and birth order of the child. As a fifth step, we control household size, wealth index, and religion in addition to the child-level factors of the previous model. In the final model, we add the regions and residence type to determine their effect on the predetermined models. We repeat this process using child wasting as the dependent variable.

## 3. Results

### 3.1. Data Sample Description

Table 1 presents a descriptive overview of the surveyed children. The sample exhibits a near-equal gender distribution, with 50.4% male and 49.6% female children. The age of the children spans a wide range, from 0 to 59 months, with each 5-month age group representing roughly 7–10% of the sample. In terms of birth order, 59.7% were within the 2nd to 5th position, while firstborn children constituted 29.37%. Larger household sizes were more prevalent, with 65.27% of households having 5–9 members.

Regarding socioeconomic status, the wealth index shows a relatively even distribution across the five quintiles, ranging from 16.55% in the poorer category to 22.20% in the richer category. Religious affiliations were diverse, with Evangelical/Pentecostal being the most common (29.46%), followed by Catholic (23.24%) and Islamic (20.55%). Geographically, the Nampula region had the largest representation (12.26%), while Cidade de Maputo had the smallest (5.90%). The majority of the children resided in rural areas (65.47%).

Access to basic amenities varied within the sample. While 65.70% had access to improved sources of drinking water, only 27.00% used improved toilet facilities. Similarly, a considerable proportion of child stool disposal was classified as not improved (29.45%). Finally, in terms of health indicators, 33.11% of the children were classified as stunted, and a higher proportion, 46.48%, were classified as wasted. We also report these characteristics by gender; distributions are similar for males and females.

### 3.2. WASH and Child Stunting

Table 2 reports odds ratios from the six adjusted logistic regression models assessing the relationship between WASH indicators and child stunting. In the unadjusted model (column 1), children in households with improved sources of water have 48% lower odds of being stunted compared to those without improved water sources (OR = 0.52, 95% CI = 0.45–0.590, *p* < 0.001). This effect persisted with minimal attenuation in columns 3–4, remaining significant through column 4 (OR = 0.54, 95% CI = 0.47–0.63, *p* < 0.001), which includes controls for sanitation, child sex, age, and birth order. However, once household size and wealth were adjusted for in column 5, the odds ratio increased to 0.77 (95% CI = 0.65–0.91) and was statistically significant at the 5% level of significance. This indicates that when child and household demographics are included in the model, children with improved water have only 33% lower chances of being stunted. In the fully adjusted model (column 6), controlling for religion, region, and residence type, the effect size further diminished (OR = 0.80, 95% CI = 0.67–0.94), though it retained statistical significance (*p* < 0.01), suggesting that improved water source has an independent but partially confounded effect on stunting risk.

The association between improved toilet facilities and stunting is nuanced and mixed. In the unadjusted model, the association between improved source of toilet and stunting is negative and statistically significant at 1% level of significance; however, this negative association initially observed between improved toilet facilities and stunting became statistically insignificant after adjusting for child, household, and socioeconomic covariates (models 5 and 6). For example, children with improved sanitation had 34% lower odds of being stunted (OR = 0.66, 95% CI = 0.56–0.78, *p* < 0.001). This association remained statistically significant in models 3 and 4, and the odds ratio increased to 0.76 (95% CI = 0.65–0.91). However, the statistically significant association between improved source of sanitation and stunting disappears after we adjust for household wealth, religion, and regions (e.g., OR = 1.15, 95% CI = 0.94–1.39) in model 5; (OR = 0.93, 95% CI = 0.76–1.13) in model 6. This suggests that the apparent protective effect of sanitation on stunting in the unadjusted models was largely explained by confounding factors such as household wealth, maternal education, and regional characteristics, rather than sanitation itself.

Female children with improved water sources and sanitation have between 32 and 34% lower odds of stunting across all models compared to male children. Child age is positively associated with stunting (OR = 1.01, 95% CI = 1.00–1.01 in columns 4–6): each additional month slightly increases odds. Birth order has no significant effect.

The wealth index remained a strong predictor: compared to the poorest quintile, children in the richer quintiles had 56% lower odds of stunting in column 5 (OR = 0.44, 95% CI = 0.33–0.56), attenuating to 46% (95% CI = 0.39–0.73) in the fully adjusted model, though both effects remained highly significant, *p* < 0.001. Regional and religious fixed effects explained additional variation, with several categories losing or gaining significance depending on the model, highlighting the importance of geographic and cultural context. Zionist (OR = 0.67, 95% CI = 0.53–0.85, *p* < 0.01) and Evangelical (OR = 0.72, 95% CI = 0.58–0.88, *p* < 0.01) households were more associated with lower odds of stunting in column 5. Households that did not identify with a religion were also less likely to have a stunted child (OR = 0.66, 95% CI = 0.48–0.88, *p* < 0.01). These significant associations were not observed in the fully controlled model (column 6), which accounted for regional and residence differences.

Households in Maputo seem to be least likely to have child stunting (OR = 0.36, 95% CI = 0.20–0.66, *p* < 0.001). Children in that region have 64% lower odds of stunting, followed by Inhambane at 57%. Contrarily, households in Manica are at greater risk of child stunting (OR = 1.77, 95% CI = 1.21–2.59, *p* < 0.01) such that children in that region have 77% greater odds of being stunted, compared to Niassa. This is also true for children in Cabo Delgado, although the odds decrease to 65%. Urban households had no significant effect.

### 3.3. WASH and Child Wasting

The results of the relationship between WASH indicators and child wasting are presented in Table 3. Children with improved water sources have 13% lower odds of wasting compared to children without access to unimproved water sources (OR = 0.87, 95% CI = 0.75–0.98, *p* < 0.05). In no other model was this association evident or statistically significant. A similar pattern was observed between improved sanitation (OR = 0.85, 95% CI = 0.74–0.99, *p* < 0.05) and child wasting. Children with improved sanitation have 15% lower odds of wasting, but such a relationship is not present in subsequent model specifications for all WASH indicators, child and household characteristics, region, and residence.

Among demographic covariates, household wealth was a weak protective factor against wasting. Children from middle-income households had substantially lower likelihoods of wasting in column 5 (OR = 0.77, 95% CI = 0.62–0.95, *p* < 0.05) compared to the poorest wealth quintile, though this effect became insignificant after regional controls.

Regions of residence were also examined as potential correlates. Children in the Zambezia region exhibited significantly higher rates of wasting (coefficient = 1.72, 95% CI = 1.22–2.41, *p* < 0.01), suggesting important geographic disparities; children in that region have 38% higher odds of wasting compared to those in Niassa. On the other hand, Cabo Delgado seemed to have the least statistically significant likelihood of child wasting (OR = 0.73, 95% CI = 0.55–0.94, *p* < 0.05), such that children in this region had 37% lower odds of wasting. No significant associations were found across most religious categories or between rural and urban households after full adjustment. Child-level variables, such as age, sex, and birth order, and household variables like religion, did not have any protective effect on wasting power.

In summary, our objective was to quantify how access to improved drinking water and improved sanitation correlated with children’s nutritional status in Mozambique—specifically, to (i) estimate associations with stunting and wasting and (ii) compare the relative contribution of water versus sanitation after accounting for confounding. Using nationally representative DHS data, we implemented a sequence of logistic regressions (water-only, sanitation-only, joint models) with progressive adjustment for child and household-level factors. Through these analyses, we achieved our objectives: improved water access shows a consistent protective association with stunting across specifications; sanitation’s association with stunting weakens after full adjustment; and neither water nor sanitation exhibits a consistent association with wasting. The analyses support the stability of these findings and the component-specific contrasts the study set out to assess.

## 4. Discussion

This study provides new national-level evidence on the role of water and sanitation in shaping child nutritional outcomes in Mozambique, a setting marked by both severe undernutrition and significant regional and infrastructural heterogeneity. Our results show that improved water access is significantly associated with reduced risk of stunting, while sanitation shows no independent effect on stunting once socioeconomic factors are controlled, and neither water nor sanitation is associated with wasting. By demonstrating that improved water access offers significant protection against stunting but not wasting, our study highlights the need to tailor interventions: clean and reliable water supplies should be prioritized to reduce chronic undernutrition, while strategies to address wasting may require direct nutritional supplementation, timely treatment of infections, and broader poverty-alleviation measures. These results offer a practical basis for prioritization: where sanitation shows the stronger association with chronic growth faltering, programs should emphasize safely managed sanitation and fecal-sludge management; where water appears more closely tied to acute undernutrition risk, efforts should focus on water-quality improvements (treatment at source/household), safe storage, and reliable service. For planning and accountability, a concise monitoring set can track coverage of basic or safely managed water and sanitation, child diarrhea prevalence, and growth indicators (e.g., HAZ/WHZ). To our knowledge, this is the first Mozambique-wide analysis that examines water and sanitation side by side for both stunting and wasting within a single framework, extending city-specific studies and providing country-level evidence to inform targeting and the sequencing of WASH with nutrition programs. While we interpret these patterns as associations rather than causal effects, they offer actionable guidance for directing scarce resources toward the WASH lever most likely to yield health gains in this context. Beyond its policy relevance, this study also makes a clear scientific contribution by using recent, nationally representative data from Mozambique to disentangle the distinct effects of water and sanitation on stunting and wasting—something that earlier studies, often based on smaller or localized samples, have not systematically addressed. By applying a stepwise regression framework, we show that the apparent associations between sanitation and child growth are largely explained by household wealth and regional characteristics, thereby offering methodological clarity to a literature where WASH components are frequently treated as a single package. This differentiation advances understanding of the biological and social pathways linking WASH to child health, while also improving the external validity of findings for program and policy design.

### 4.1. Improved Water Access, Child Stunting, and Wasting

We find a persistent and statistically significant association between improved access to drinking water and reduced odds of child stunting, even after adjusting for household wealth, region, religion, and other sociodemographic factors. This aligns with findings in Indonesia [7] and contests findings of a study in Karamoja, Uganda [23]. The effect is more pronounced for stunting than for wasting, likely due to the chronic nature of stunting and its stronger biological links to repeated enteric infections from unsafe water to impaired nutrient absorption over time [2,24]. However, the attenuation of the effect size with full adjustment suggests that clean water is not universally transformative. Its impact depends on the consistency of use, household storage practices, and whether clean water is paired with adequate hygiene behaviors [25]. Thus, policies should not interpret “improved source” categorization as a sufficient indicator of effective intervention. Rather, investment in water infrastructure must be integrated with sustained behavior change programming, routine quality monitoring, and tailored community health education that reflects local context and norms.

### 4.2. Improved Sanitation, Child Stunting, and Wasting

Improved sanitation was not significantly associated with either stunting or wasting after adjusting for household-level and regional variables. These findings temper the conclusion that improved sanitation had meaningfully reduced the risk of wasting in Laos [12] but agree with findings in Benin, where there was no evidence of a significant relationship between household access to WASH and wasting in children under five [11]. These patterns are consistent with biological theory: stunting reflects chronic nutritional stress and long-term exposures, conditions shaped by sustained water quality and infection burden, while wasting is often a short-term response to acute food insecurity or illness. In addition, households may have improved toilets but not use them consistently, or may live in communities where open defecation persists at the population level, undermining household-level gains. Indeed, some studies suggest that unimproved toilet facilities and unprotected water sources are better predictors of wasting than the presence of improved sanitation per se [26]. Notable nutritional gains from sanitation may require threshold effects, where benefits accrue only when usage reaches a critical density across communities.

For policymakers, it suggests that WASH interventions alone may not be the best solution to reduce wasting in children under five in Mozambique and that alternatives or better integrated WASH programs are needed.

### 4.3. Gender, Religion, and Child Malnutrition

This study also highlights subtler but equally important sources of variation. Female children had significantly higher odds of stunting compared to male children, in contrast to global patterns where boys are typically more vulnerable to undernutrition [27]. This reversal may reflect gendered caregiving norms in Mozambique that prioritize male children in intra-household food allocation or health-seeking behavior. If so, standard nutritional interventions will be insufficient unless they also address underlying social norms. Similarly, religion, often excluded from policy discussions, was associated with stunting risk in some models. Though effects attenuated after full adjustment, the consistent associations with Zionist and Evangelical households align with the idea that different religious affiliations may influence child health. For instance, the improved child survival seen among Catholic and mainline Protestant or Evangelical families is likely linked to these churches’ stronger integration with the healthcare system, while the positive outcomes associated with Apostolic/Zionist households may stem from close-knit social networks and mutual support within their congregations [28]. This suggests that faith-based beliefs and networks may shape health behaviors, caregiving practices, or even food availability.

### 4.4. Wealth, Regional Disparities, and Child Malnutrition

Children in Maputo had significantly lower odds of stunting and Cabo Delgado of wasting, while children in Manica and Zambezia had the highest odds of stunting and wasting, respectively. Children in Maputo, an urban region, have significantly lower rates of stunting and wasting, possibly due to stronger economic integration, particularly through proximity to South Africa. As such, mothers and their children have better access to healthcare services [29]. In contrast, children in Manica and Zambezia face higher risks due to widespread poverty and limited access to these services. This supports our findings that having a stable socioeconomic status (higher household wealth) was positively associated with decreased risk of stunting and wasting, although for wasting, being in the middle class was enough to see this relationship. In regions such as Niassa, Cabo Delgado, Sofala, Zambezia, and Tete, maternal employment was significantly associated with lower rates of stunting among children under five [30]. This protective effect is also attributed to increased household income, improved access to healthcare, and enhanced decision-making power within households, all of which contribute to better child nutrition outcomes.

National policies that allocate resources uniformly across provinces may miss these concentrated burdens. A more efficient and equitable approach would direct WASH investments according to regional risk profiles, prioritizing water infrastructure and behavioral programming in high-stunting regions, while deploying rapid-response nutrition and disease-prevention interventions in regions with acute wasting risk. Public health strategies could leverage these institutions as partners in behavior change, while also investigating whether certain religious norms inadvertently reinforce nutritional disadvantage.

Taken together, these findings challenge reductionist approaches to WASH policy. Improved infrastructure is only one part of a much broader puzzle. To maximize nutritional impact, WASH investments must be layered with interventions that target behavior, community norms, and systemic inequities, particularly poverty, maternal education, and regional access to services. Our findings also argue against universalist assumptions: the impact of WASH varies not only by component (water vs. sanitation), but by geography, gender, and religion. Public health policies should move toward tailored, multi-sectoral strategies that reflect these nuances.

### 4.5. Limitations

The study has a few limitations. First, low birthweight or pre-term births could be an important predictor of stunting/wasting. The statistical model should adjust for these measures of birth outcomes. However, the Mozambique DHS data measures birthweight only for a subset of children (primarily when a health card is available or based on the mother’s recall), resulting in substantial missingness and reduced sample size. Moreover, birthweight/prematurity may lie on the causal pathway from WASH to child growth, raising concerns about post-treatment adjustment. We therefore did not include birthweight or prematurity in our empirical models. Second, the binary categorization of “improved” vs. “unimproved” infrastructure may obscure substantial heterogeneity in usage, quality, and reliability. Low R-squared values across models suggest that many relevant determinants—such as dietary quality, food security, maternal mental health, and intra-household dynamics—remain unmeasured. Nonetheless, the analytic strength of this study lies in its ability to isolate specific WASH pathways while controlling multiple household and regional covariates. Third, maternal education and health have been associated with child malnutrition, but were not evaluated in this paper due to significant missing values.

## 5. Conclusions

In conclusion, our findings indicate that improving access to safe drinking water is the WASH lever most closely linked to lower stunting, whereas sanitation shows no independent association with stunting, and neither water nor sanitation is associated with wasting. Policy should therefore prioritize drinking-water investments, treatment at source and household level, safe storage, service reliability, and protection of supply points, especially in high-burden provinces and among poorer households. By contrast, reducing wasting will require complementary measures beyond WASH (e.g., timely identification and treatment of acute malnutrition, infection prevention and case management, food security and social protection, and behavior change for infant and young child feeding). Programmatically, ministries can sequence: (1) expand safely managed/basic water coverage and water-quality monitoring where stunting is highest; (2) maintain sanitation upgrades for broader health and dignity benefits, while not expecting short-run growth gains; and (3) track a concise dashboard (water/sanitation coverage, water quality, diarrhea prevalence, HAZ/WHZ). These are associative findings, not causal effects, and future work should examine service quality (microbial water tests, continuity), sanitation safely managed metrics (containment/emptying), and longitudinal designs to test mechanisms. Still, by separating water from sanitation in nationally representative data, the study provides actionable guidance for targeting limited resources to the WASH components most likely to yield child growth benefits in Mozambique.

## Figures and Tables

**Table 1:** Descriptive Statistics (Analytical Sample; N = 3690).

Variable	Category	Total Sample (%)	Male (%)	Female (%)
Stunting	Yes	33.06	37.17	28.97
	No	66.94	62.83	71.03
Wasting	Yes	46.50	46.47	46.54
	No	53.50	53.53	53.46
Source of drinking water	Improved	62.30	62.83	61.78
	Not improved	37.70	37.17	38.22
Toilet facilities	Improved	25.91	26.30	25.51
	Not improved	74.09	73.70	74.49
Gender of child	Male	50.1	-	-
	Female	49.9	-	-
Age of child (months)	0–11	22.7	22.07	23.41
	12–23	20.2	19.89	20.59
	24–35	20.6	19.46	21.73
	36–59	36.5	38.59	34.27
Birth order	1	23.66	24.51	22.81
	2–5	61.08	60.54	61.62
	6–9	14.23	14.02	14.43
	10–15	1.03	0.92	1.14
Household size	0–4	25.09	25.38	24.81
	5–9	63.60	63.59	63.62
	10–14	11.31	11.03	11.57
Wealth index	Poorest	21.17	19.46	22.86
	Poorer	18.81	19.29	18.32
	Middle	23.17	23.75	22.59
	Richer	20.35	20.60	20.11
	Richest	16.50	16.90	16.11
Religion	Catholic	23.96	24.62	23.3
	Islamic	24.15	23.70	24.59
	Zion	14.77	15.05	14.19
	Evangelical/Pentecostal	27.15	27.23	27.08
	Anglican	2.14	2.07	2.22
	No religion	7.18	6.79	7.57
	Other	0.65	0.5	0.76
Region	Niassa	12.41	12.66	12.16
	Cabo Delgado	13.33	14.02	12.65
	Nampula	13.66	13.15	14.16
	Zambezia	6.67	6.25	7.08
	Tete	9.35	7.93	10.76
	Manica	10.84	11.58	10.11
	Sofala	10.35	9.84	10.86
	Inhambane	6.42	6.41	6.43
	Gaza	7.07	7.39	6.76
	Maputo	5.53	5.87	5.19
	Cidade de Maputo	4.36	4.89	3.84
Type of residence	Urban	31.38	31.85	30.92
	Rural	68.62	68.15	69.08

**Table 2:** Adjusted odds ratios and confidence intervals from logit regression models examining the association between WASH indicators and stunting among children under five.

	**Model 1**	**Model 2**	**Model 3**	**Model 4**	**Model 5**	**Model 6**
Improved water source	0.52 ***		0.55 ***	0.54 ***	0.77 **	0.80 **
	(0.45–0.59)		(0.47–0.63)	(0.47–0.63)	(0.65–0.91)	(0.67–0.94)
Improved toilet facilities		0.66 ***	0.76 ***	0.76 ***	1.15	0.93
		(0.56–0.78)	(0.65–0.91)	(0.64–0.91)	(0.94–1.39)	(0.76–1.13)
Female				0.68 ***	0.66 ***	0.66 ***
				(0.59–0.78)	(0.58–0.77)	(0.57–0.76)
Child age (months)				1.01 ***	1.01 ***	1.01 ***
				(1.00–1.01)	(1.00–1.01)	(1.00–1.01)
Birth order				1.03 *	1.00	1
				(0.99–1.07)	(0.97–1.04)	(0.96–1.03)
Household size					0.99	0.99
					(0.97–1.02)	(0.97–1.02)
Wealth groups						
Poorest (reference)					-	-
Poorer					0.96	0.95
					(0.77–1.18)	(0.76–1.17)
Middle					0.83 *	0.91
					(0.67–1.03)	(0.72–1.14)
Richer					0.44 ***	0.54 ***
					(0.33–0.56)	(0.39–0.73)
Richest					0.22 ***	0.33 ***
					(0.16–0.31)	(0.22–0.48)
Religion						
Catholic (reference)					-	-
Islamic					0.96	0.87
					(0.78–1.16)	(0.70–1.07)
Zion					0.67 ***	0.84
					(0.53–0.85)	(0.61–1.15)
Evangelical					0.72 ***	0.93
					(0.58–0.88)	(0.71–1.21)
Anglican					1.14	0.99
					(0.69–1.84)	(0.58–1.68)
No religion					0.66 ***	0.72 *
					(0.48–0.88)	(0.50–1.01)
** ** Other					0.41	0.35 *
					(0.13–1.25)	(0.11–1.07)
Regions						
Niassa (reference)						-
Cabo Delgado						1.65 ***
						(1.24–2.17)
Nampula						1.61 ***
						(1.23–2.11)
Zambezia						1.17
						(0.82–1.65)
Tete						1.33
						(0.93–1.90)
Manica						1.77 ***
						(1.21–2.59)
Sofala						1.01
						(0.69–1.46)
Inhambane						0.43 ***
						(0.26–0.68)
Gaza						0.58 **
						(0.36–0.94)
Maputo						0.36 ***
						(0.20–0.66)
Cidade de Maputo						0.56 *
						(0.29–1.03)
Urban households						1.06
						(0.84–1.34)
N	3690	3690	3690	3690	3690	3690

Notes: Stepwise logistic regression models of the association between improved water and sanitation facilities and child stunting in Mozambique. Model 1 shows the independent, unadjusted effect of improved water on stunting. Model 2 shows the independent, unadjusted effect of improved sanitation on stunting. Model 3 includes both water and sanitation simultaneously. Model 4 adjusts for child characteristics and household size. Model 5 adds household wealth and religion. Model 6 is the fully adjusted model, including all covariates. Odds ratios (ORs) and 95% confidence intervals (CIs) are reported. CIs are reported in parentheses. * *p* < 0.01; ** *p* < 0.05; *** *p* < 0.10.

**Table 3:** Adjusted odds ratios and confidence intervals from logit regression models examining the association between WASH indicators and wasting among children under five.

	Model 1	Model 2	Model 3	Model 4	Model 5	Model 6
Improved water source	0.87 **		0.89 *	0.89 *	0.96	1.01
	(0.75–0.98)		(0.77–1.02)	(0.77–1.02)	(0.82–1.11)	(0.86–1.18)
Improved toilet facilities		0.85 **	0.88 *	0.88	0.92	0.88
		(0.74–0.99)	(0.75–1.02)	(0.76–1.02)	(0.77–1.08)	(0.74–1.04)
Female				1.00	0.99	0.97
				(0.88–1.14)	(0.87–1.13)	(0.85–1.11)
Child age (months)				1.00 *	1.00	1.00 *
				(0.99–1.01)	(0.99–1.01)	(0.99–1.01)
Birth order				1.01	1.00	1.01
				(0.98–1.04)	(0.97–1.04)	(0.97–1.04)
Household size					1.01	1.01
					(0.98–1.03)	(0.98–1.03)
Wealth groups						
Poorest (reference)					-	-
Poorer					0.88	0.89
					(0.72–1.08)	(0.72–1.10)
Middle					0.77 **	0.85
					(0.62–0.95)	(0.68–1.05)
Richer					0.81 *	0.93
					(0.64–1.01)	(0.71–1.21)
Richest					0.8	1
					(0.62–1.04)	(0.72–1.39)
Religion						
Catholic (reference)					-	-
Islamic					1.06	1.16
					(0.87–1.28)	(0.94–1.42)
Zion					0.84	0.9
					(0.67–1.04)	(0.68–1.17)
Evangelical					1	1.01
					(0.83–1.20)	(0.80–1.27)
Anglican					0.98	1.16
					(0.62–1.54)	(0.69–1.93)
No religion					1.18	1.17
					(0.89–1.56)	(0.85–1.59)
Other					1.63	1.71
					(0.71–3.74)	(0.72–4.03)
Regions						
Niassa (reference)						-
Cabo Delgado						0.73 **
						(0.55–0.94)
Nampula						1.1
						(0.85–1.42)
Zambezia						1.72 ***
						(1.22–2.41)
Tete						1.11
						(0.79–1.54)
Manica						0.75
						(0.53–1.06)
Sofala						1.12
						(0.79–1.56)
Inhambane						0.93
						(0.63–1.35)
Gaza						0.91
						(0.62–1.34)
Maputo						0.71
						(0.47–1.07)
Cidade de Maputo						0.91
						(0.58–1.41)
Urban households						0.9
						(0.74–1.09)
N	3690	3690	3690	3690	3690	3690

Notes: Stepwise logistic regression models of the association between improved water and sanitation facilities and child wasting in Mozambique. Model 1 shows the independent, unadjusted effect of improved water on wasting. Model 2 shows the independent, unadjusted effect of improved sanitation on wasting. Model 3 includes both water and sanitation simultaneously. Model 4 adjusts for child characteristics and household size. Model 5 adds household wealth and religion. Model 6 is the fully adjusted model, including all covariates. Odds ratios (ORs) and 95% confidence intervals (CIs) are reported. CIs are reported in parentheses. * *p* < 0.01; ** *p* < 0.05; *** *p* < 0.10.

## Data Availability

All data used for this study are publicly available on the DHS portal. The data is de-identified and accessible to researchers upon request through the DHS Program website (https://dhsprogram.com, accessed on 20 January 2025), and may be subject to registration and approval of a brief research proposal.
