# Correction: Castillo et al. Water Matters More: Unequal Effects of Water and Sanitation on Child Growth in Mozambique. *Children* 2025, *12*, 1414

**DOI:** 10.3390/children12111536

**Published:** 2025-11-14

**Authors:** Jailene P. Castillo, Christina A. Molinaro, William E. Pater, Santosh K. Gautam

**Affiliations:** 1 Keough School of Global Affairs, University of Notre Dame, Notre Dame, IN 46556, USA; 2 Eck Institute for Global Health, University of Notre Dame, Notre Dame, IN 46556, USA; 3 Department of Biological Sciences, University of Notre Dame, Notre Dame, IN 46556, USA

## Table Legend

In the original publication [1], there was a mistake in the legend for Table 2, adjusted odds ratios and confidence intervals from logit regression models examining the association between WASH indicators and stunting among children under five, and Table 3, adjusted odds ratios and confidence intervals from logit regression models examining the association between WASH indicators and wasting among children under five.

The order of *p*-value significance was reversed—* *p* < 0.01; ** *p* < 0.05; *** *p* < 0.10.

The correct legend is as follows: * *p* < 0.10; ** *p* < 0.05; *** *p* < 0.01.

## Text Correction

In the published publication [1], the summary statistic percentages reported in subsection 3.1. Data Sample Description, paragraphs 1–3, do not match the percentages reported in Table 1. Descriptive Statistics (Analytical Sample; N = 3690).

A correction has been made to subsection 3.1. Data Sample Description, paragraph 1:

“Table 1 presents a descriptive overview of the surveyed children. The sample exhibits a near-equal gender distribution, with 50.1% male and 49.9% female children. The age of the children spans a wide range, from 0 to 59 months: 22.7% are 0–11 months old, 20.2% are 12–23 months old, 20.6% are 24–35 months old, and 36.5% are 36–59 months old. In terms of birth order, 61.1% were within the 2nd to 5th position, while firstborn children constituted 23.66%. Larger household sizes were more prevalent, with 63.6% of households having 5–9 members.”

A correction has been made to subsection 3.1. Data Sample Description, paragraph 2:

“Regarding socioeconomic status, the wealth index shows a relatively even distribution across the five quintiles, ranging from 16.5% in the richest category to 23.17% in the middle category. Religious affiliations were diverse, with Evangelical/Pentecostal being the most common (27.15%), followed by Catholic (23.96%) and Islamic (24.15%). Geographically, the Nampula region had the largest representation (13.66%), while Cidade de Maputo had the smallest (4.36%). The majority of the children resided in rural areas (68.62%).”

A correction has been made to subsection 3.1. Data Sample Description, paragraph 3:

“Access to basic amenities varied within the sample. While 62.3% had access to improved sources of drinking water, only 26% had access to improved toilet facilities. Finally, in terms of health indicators, 33.06% of the children were classified as stunted, and a higher proportion, 46.5%, were classified as wasted. We also report these characteristics by gender; distributions are similar for males and females.”

We also corrected a few typos throughout the manuscript.

1. Introduction, paragraph 1:
  - Replaced low- and middle-income countries with low- and middle-income countries (LMICs)
  - Replaced height-for-age with height-for-age z-score (HAZ)
  - Replaced WHO with World Health Organization (WHO)
  - Replaced weight for height with weight-for-height z-score (WHZ)
  - Replaced Sub-Saharan Africa with Sub-Saharan Africa (SSA)
  - Replaced Sub-Saharan Africa with SSA
1. Introduction, paragraph 6:
  - Replaced low- and middle-income countries with LMICs
2.6. Statistical Analysis:
  - Replaced Stata with STATA
  - Replaced models with outcome variables
3.2. WASH and Child Stunting, paragraph 2:
  - Replaced sanitation with sanitation in column 2

The authors state that the scientific conclusions are unaffected. This correction was approved by the Academic Editor. The original publication has also been updated.
